# Le shwannome extrathoracique solitaire du nerf intercostal: une localisation rare

**DOI:** 10.11604/pamj.2014.18.178.315

**Published:** 2014-06-24

**Authors:** Amal Ankouz, Abdellatif Louchi, Khalid Ait Taleb

**Affiliations:** 1 Service de chirurgie digestive C3, CHU Hassan II de Fès, Maroc

**Keywords:** Schwannome extra-thoracique, nerf intercostal, chirurgie, Extra-thoracic schwannoma, intercostal nerve, surgery

## Abstract

Le shwannome thoracique solitaire est une tumeur nerveuse bénigne. La localisation est le plus souvent intra-thoracique au niveau du médiastin postérieur. Nous rapportons l'observation d'un schwannome solitaire du nerf intercostal de développement extra-thoracique imitant une tumeur sous-cutanée du thorax. La biopsie-exérèse nous a permis d’établir le diagnostic histologique. Après un recul de 14 mois aucune récidive n'a été notée. Le diagnostic de schwannome doit être suspecté devant toute tumeur sous cutanée du thorax. La certitude diagnostique est histologique. L'exérèse totale est le seul garant d'une guérison sans récidive.

## Introduction

Le shwannome est une tumeur nerveuse bénigne qui se développe à partir de la gaine de la cellule de schwan. Il représente la plus fréquente des tumeurs nerveuses du thorax. La localisation est le plus souvent intra-thoracique au niveau du mediastin postérieur. Nous rapportons l'observation d'un schwannome extra-thoracique imittant une tumeur sous-cutané du thorax.

## Patient et observation

**Figure 1:**
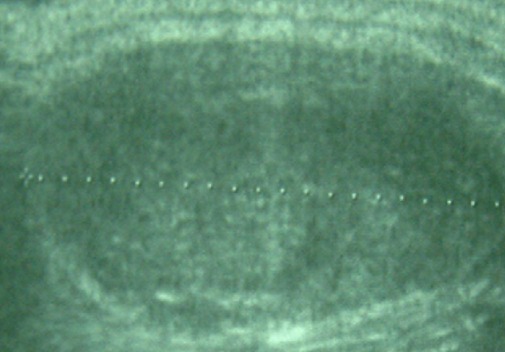
Aspect échographique d'une masse sous-cutanée bien limitée hypoéchogène, homogène, mesurant 2,4 cm de grand axe

E.K, âgée de 50 ans, sans antécédent pathologique notable, consulte pour une tuméfaction asymptomatique de la face latérale sur la ligne axillaire moyenne de l'hemithorax droit en regard du dixième espace intercostal droit évoluant depuis deux ans et augmentant progressivement de volume. L'examen clinique trouvait une tuméfaction ovalaire de 2cm de grand diamètre, dure, mobile par rapport au plan profond, indolore à la palpation. La peau en regard était normale. Par ailleurs, l'examen général n'objectivait pas d'autre tuméfaction ni de signes orientant vers une maladie de recklinghausen. L’échographie trouvait une masse sous-cutanée bien limitée hypoéchogène, homogène, mesurant 2,4 cm de grand diamètre, faisant évoquer un lipome sous-cutané (Figure 1). Une biopsie-exérèse était indiquée. L'exploration chirurgicale était faite sous anesthésie locale et sans aucun moyen de grossissement. La dissection de la tumeur était aisée. Il s'agissait d'une tumeur encapsulée, ferme, de couleur jaunatre, localisée dans le tissu sous-cutané. En peropératoire, on ne notait pas de rapport avec des éléments nerveux individualisables.

**Figure 2:**
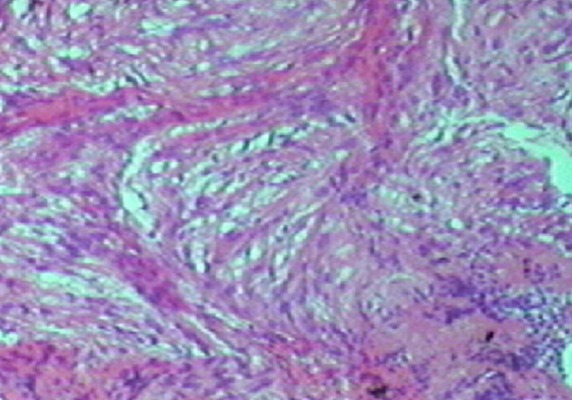
Aspect histologique évoquant un schwannome bénin

L’étude anatomopathologique de la pièce trouvait une prolifération tumorale bénigne multinodulaire entourée de capsules fibreuses. Les nodules étaient faits de cellules fusiformes de densité variable se disposant en faisceaux courts entrecroisés avec des noyaux hyperchromatiques réguliers se disposant en palissades, réalisant par endroit des images d'enroulement correspondant au corps de Verocay; le fond était myxoïde et lâche (Figure 2). L’étude immunohistochimique utilisant l'anticorps monoclonal PS100 avait très positivement marqué les cellules tumorales.

Le diagnostic de schwannome extra thoracique a été retenu. Nous avons déduit qu'il s'agissait d'un schwannome aux dépends du bout distal d'un fin rameau nerveux superficiel issu du nerf intercostal. La patiente a été revue à deux ans de recul sans aucune récidive.

## Discussion

La majorité des tumeurs primitives thoraciques sont malignes, seulement 20% sont bénignes [1]. Dans le médiastin postérieur, 63% des tumeurs sont neurogènes [2], parmi lesquelles les schwannomes bénins sont les plus fréquents chez l'adulte [3]. Dans 5% des cas, Ils se développent généralement à partir des nerfs intercostaux du médiastin postérosupérieur (beaucoup plus rarement à partir des nerfs vague, phrénique ou récurrent [2–4]. Leur extension ultérieure peut se faire dans la plèvre pariétale et médiastinale, épargnant habituellement la plèvre viscérale [4]. Les autres localisations fréquentes du schwannome sont: le cou, l'extrémité des membres et l'angle ponto-cérébelleux.

Rarement que le développement du schwannome ne se fera en périphérie en dehors de la cavité thoracique. Après une revue de la littérature internationale, Hiroyuki a rapporté le premier cas de neurolinome sous cutané du nerf intercostal, aucun autre cas n'a été signalé. Notre observation semble être la deuxième [5].

Ce mode de développement superficiel en extrathoracique du schwannome peut être expliqué par une anomalie anatomique du nerf intercostal. Normalement, le nerf intercostal se trouve le plus souvent avec les vaisseaux intercostaux le long de la gouttière costale, étant situé dans la limite supérieure de l'espace intercostal. Dans le cas rapporté par Hiroyuki, le nerf se situait probablement plus loin de cette limite [5]. Cependant, dans notre cas, aucune structure nerveuse n'a été individualisée, nous avons déduit qu'il s'agissait d'un schwannome aux dépends de l'extrémité distale d'un fin rameau nerveux perforant issu du nerf intercostal.

Le schwannome est une tumeur bénigne encapsulée qui prolifère à partir de la gaine des cellules de schwann. Généralement, c'est une tumeur solitaire. Toutefois, des localisations multiples peuvent s'intégrer dans le cadre d'une maladie de Reckling Haussen [6, 7]

L'IRM peut orienter le diagnostic en objectivant un aspect en faveur d'un schwannome. Elle peut montrer en T1 un signal de même intensité ou légèrement supérieur au muscle, en T2 un signal hyperintense parfois une « image en cible » avec un halo périphérique hyperintense et un centre hypo-intense [8]. La confirmation du diagnostic reste histologique.

Le diagnostic différentiel se fait essentiellement avec les autres tumeurs neurogènes: neurofibromes, neurofibrosarcome et schwannosarcome [3].

Le traitement de choix est l'exérèse chirurgicale puisque il s'agit d'une tumeur facilement extirpable. Après exérèse complète, il n'y a pas de récidive. La dégénérescence maligne d'un schwannome en schwannome malin est controversée, une méconnaissance du diagnostic de malignité initial ne peut être écartée [9–11]. En ce qui concerne les schwannomes isolés ou même les schwannomes multiples de la schwannomatose, aucun cas de dégénérescence maligne n'a été décrit actuellement [9, 12].

Dans notre cas, l'exérèse était complète sans aucune récidive locale.

## Conclusion

Le schwannome solitaire du nerf intercostal avec un mode de développement extra-thoracique reste exceptionnel. Le diagnostic doit être suspecté devant toute tumeur sous cutanée du thorax. La certitude diagnostique est histologique. L'exérèse totale est le seul garant d'une guérison sans récidive.
